# Depression and other correlates of adult Attention Deficit Hyperactivity Disorder (ADHD) symptoms among Hungarian university students

**DOI:** 10.1192/j.eurpsy.2022.1159

**Published:** 2022-09-01

**Authors:** V. Müller, B. Pikó

**Affiliations:** 1 University of Szeged, Doctoral School Of Education, Szeged, Hungary; 2 University of Szeged, Department Of Behavioral Sciences, Szeged, Hungary

**Keywords:** adhd, Depression, resilience, life satisfaction

## Abstract

**Introduction:**

Attention Deficit Hyperactivity Disorder (ADHD) is a neurodevelopmental disorder characterized by symptoms of inattention, hyperactivity, and/or impulsivity. It is one of the most common disabilities in college populations and comorbidity with depression is frequently reported.

**Objectives:**

The aim of the study is to shed light on depression as comorbidity and other intrapersonal correlates of ADHD in young adults.

**Methods:**

Participants were Hungarian university students (N=420; M=24.5, SD=5.0 years). Criteria of the ADHD group were based on the Adult ADHD Self Report Scale V1.1 (ASRS-V.1.1) screening tool. The participants filled in the Beck’s Depression Inventory, the Hyperfocus Scale, Flow State Scale, Academic Persistence Scale, Satisfaction With Life Scale, General Self-Efficacy Scale, and the Connor-Davidson Resilience Scale.

**Results:**

We found that in the group of students who had ADHD symptoms, depression score was significantly (p<.001) higher (M=18.38, SD=5.87) than the control group’s scores (M=14.56, SD=4.45). Frequency of severe depression was 13.4% (moderate: 33.5%) while in the control group: 1.6% and 17.6% respectively. Participants reporting ADHD symptoms (N=164, 39%) also reported lower levels of resilience (M=23.40, SD=6.96), relative to their non-ADHD peers (M=27.69, SD=6.48). Significant differences were found in the areas of self-efficacy, depression, flow and hyperfocus as well, and ADHD symptoms contributed to lower level of life satisfaction (β=-0.24, p<.001).

**Conclusions:**

Our findings suggest that university students reporting symptoms of ADHD may be assisted with strategies that are focused on increasing protective factors (i.e., resilience, self-efficacy, flow) to prevent depression and improve their life satisfaction and quality of life.

**Disclosure:**

No significant relationships.

